# The binding capability of plasma phospholipid transfer protein, but not HDL pool size, is critical to repress LPS induced inflammation

**DOI:** 10.1038/srep20845

**Published:** 2016-02-09

**Authors:** Yang Yu, Yingjie Cui, Yanan Zhao, Shuai Liu, Guohua Song, Peng Jiao, Bin Li, Tian Luo, Shoudong Guo, Xiangjian Zhang, Hao Wang, Xian-Cheng Jiang, Shucun Qin

**Affiliations:** 1 Key Laboratory of Atherosclerosis in Universities of Shandong and Institute of Atherosclerosis, Taishan Medical University, Taian, China, 271000; 2 Department of Anatomy and Cell Biology, SUNY Downstate Medical Center, New York, USA, 11203; 3 Hebei Collaborative Innovation Center for Cardio-cerebrovascular Disease and Hebei Key Laboratory of Vascular Homeostasis, Shijiazhuang, China, 050000; 4 School of Basic Medicine, Taishan Medical University, Tai’an, China, 271000

## Abstract

Phospholipid transfer protein (PLTP) participates in high density lipoprotein (HDL) metabolism. Increased plasma PLTP activity was observed in lipopolysaccharide (LPS) triggered acute inflammatory diseases. This study aimed to determine the exact role of PLTP in LPS induced inflammation. HDL pool size was shrunk both in PLTP deficient mice (PLTP−/−) and PLTP transgenic mice (PLTP-Tg). PLTP displayed a strong protective effect on lethal endotoxemia in mice survival study. Furthermore, after LPS stimulation, the expression of pro-inflammatory cytokines were increased in bone marrow derived macrophage (BMDM) from PLTP−/−, while decreased in BMDM from PLTP-Tg compared with BMDM from wild-type mice (WT). Moreover, LPS induced nuclear factor kappa-B (NFκB) activation was enhanced in PLTP−/− BMDM or PLTP knockdown RAW264.7. Conversely, PLTP overexpression countered the NFκB activation in LPS challenged BMDM. Additionally, the activation of toll like receptor 4 (TLR4) induced by LPS showed no alteration in PLTP−/− BMDM. Finally, PLTP could bind to LPS, attenuate the pro-inflammatory effects of LPS, and improve the cell viability *in vitro*. To sum up, these findings elucidated that PLTP repressed LPS induced inflammation due to extracellular LPS binding capability, and the protective effects were not related to HDL pool size in mice.

Lipopolysaccharide (LPS), the dominant structural component of Gram-negative bacteria outer membrane, initiates strong inflammatory responses in many infectious diseases. An excessive circulatory LPS mediated high level of pro-inflammatory cytokines secretion causes lethal endotoxemia^1^. The key therapeutic strategy of endotoxemia includes the promotion of LPS elimination from circulation and the suppression of inflammatory responses mediated by immune cells^1^,^2^. High density lipoprotein (HDL) is the main carrier to transport LPS for elimination from circulation via liver during endotoxemia or sepsis^2^,^3^. Previous researches suggesting that the augment of HDL is associated with an attenuation of LPS-triggered inflammatory response strongly support the hypothesis that raising plasma HDL may characterize a therapeutic approach in the treatment of endotoxemia and its complications^4^,^5^. The story, however, is more complicated, and not only HDL particles, but also, plasma proteins involving HDL metabolism are decisive.

Phospholipid transfer protein (PLTP) is one of the proteins mediating HDL metabolism and remodeling^2^,^6^,^7^. Moreover, PLTP belongs to the members of positive acute-phase reactants with a potential role of innate immune^2^. Increased plasma PLTP activity was reported in patients with severe sepsis^8^,^9^, periodontitis^10^, and other acute or chronic inflammatory diseases^11^. Systematic PLTP deficient mice (PLTP−/−) showed moderate responses to interleukin-6 (IL-6) stimulation, whereas lethal endotoxemia caused a higher expression of pro-inflammatory cytokines, stronger organ damage, and lower survival rate in PLTP−/−, demonstrating that PLTP is a necessary protective protein in LPS induced inflammatory responses^12^,^13^. Recombinant PLTP (5 μg/ml) showed anti-inflammatory effects by neutralizing LPS and activating macrophage ATP binding cassette transporter A1 (ABCA1)- Janus kinase-2 (JAK2)- signal transducer and activator of transcription 3 (STAT3) pathways^6^,^14^, suggesting that extracellular effects might be the major manner of PLTP involved in LPS induced inflammatory responses. However, PLTP is still reported as a transporter which facilitates LPS clearance via HDL catabolism pathway.

The major plasma contributor to PLTP activity is macrophage, which plays critical roles in LPS induced inflammatory responses by involving the initiation or restraint of inflammation^15^. The release of anti-inflammatory cytokines and certain protective proteins is one of the major functions of activated macrophage needed to repress inflammatory responses^16^. Considering the increasing plasma PLTP activity in patients with endotoxemia or sepsis, we hypothesized that PLTP play key protective roles in LPS induced inflammatory responses. In the present study, we investigated the effects of PLTP expression on mice survival rate of lethal endotoxemia and the role of PLTP on LPS induced NFκB activation in macrophage.

## Results

### Plasma PLTP activity is critical to survival in lethal endotoxemia in mice

**Figure 1:**
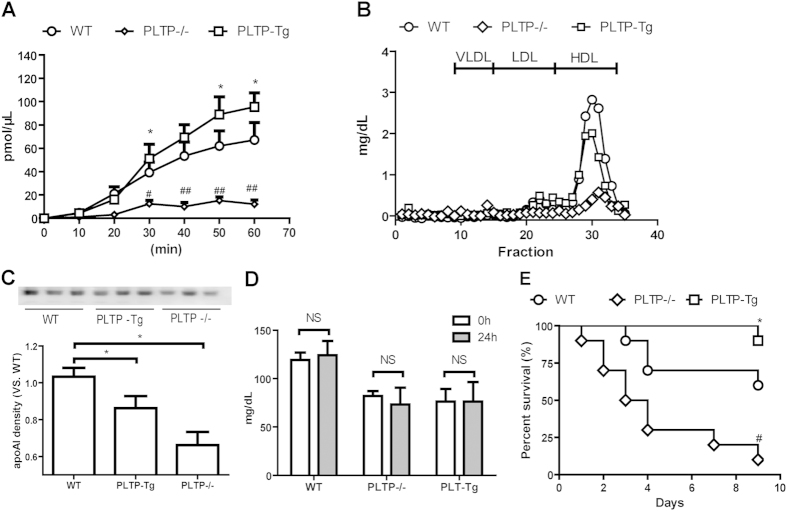
The role of PLTP on endotoxic shock status in mice. **(A**) Before LPS injection, plasma PLTP activity of wild type mice (WT, N = 5), PLTP transgenic mice (PLTP-Tg, N = 5), and PLTP knockout mice (PLTP−/−, N = 5) were determined through the fluorescent phospholipids transferring from donor to acceptor particles (See method for detail).*P < 0.05 for PLTP-Tg VS. WT; ^#^P < 0.05 for PLTP−/− VS. WT. (**B**) Pooled plasma (100 μL) from 4 or 5 mice of each genotype were fractionated by fast protein liquid chromatography (FPLC). Cholesterol level were determined and plotted as a function of FPLC fractions. The fractions containing lipoproteins are indicated. (**C**) Upper panel: plasma apoAI levels from PLTP−/−, WT, or PLTP-Tg were detected by western blot (WB). Lower panel: Densitometric quantitation of WB (n = 4), *P < 0.05 VS. WT. **P < 0.01 VS. WT. **(D**) HDL-C levels were assayed before and after LPS intraperitoneal injection. (WT, 6 mg/Kg, N = 10; PLTP-Tg, 6 mg/Kg, N = 10; PLTP−/−, 5 mg/Kg, N = 10.) (**E**) Compared with WT, PLTP-Tg showed a higher survival rate after LPS injection for 9 days, while PLTP−/− displayed nearly 10% survival individuals after LPS injection. Survival rates were analyzed by the Kaplan-Meier method and compared using the *X*^2^ test. *P < 0.05 for PLTP-Tg VS. WT; ^#^P < 0.05 for PLTP−/− VS. WT.

Plasma PLTP activity and HDL were determined in PLTP transgenic mice (PLTP-Tg), wild type mice (WT), and PLTP−/− prior to LPS injection. Plasma PLTP activity was almost absent in PLTP−/−, while moderately enhanced in PLTP-Tg compared with WT. (Fig. 1A) As is shown in Fig. 1B,C, the cholesterol level and the apoAI level of HDL were decreased both in PLTP-Tg and PLTP−/− compared with WT. Twenty-four hours after 6 mg/kg LPS injection (5 mg/kg in PLTP−/−), HDL cholesterol (HDL-C) levels were not changed.(Fig. 1D) Nine days after LPS injection, the survival rates were 90%, 60%, and 10% in PLTP-Tg, WT, and PLTP−/−, respectively. (Fig. 1E) The survival rate was closely related to plasma PLTP activity, strongly indicating that plasma PLTP was the key protective factors to mice survival in lethal endotoxemia. Although HDL pool size is considered as the decisive factor in LPS clearance *in vivo*, our findings suggested that PLTP other than HDL pool size is the primary cause to maintain the survival of mice in lethal endotoxemia.

### PLTP exerts anti-inflammatory effects in lethal endotoxemia

**Figure 2:**
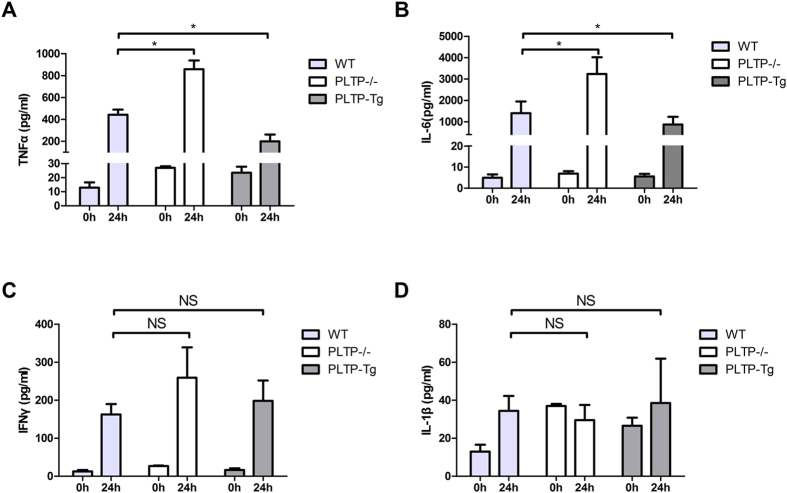
The effects of PLTP on inflammatory cytokines in lethal endotoxemia. Before and 24 hours after LPS injection,(WT, 6 mg/Kg, N = 10; PLTP-Tg, 6 mg/Kg, N = 10; PLTP−/−, 5 mg/Kg, N = 10). The plasma were isolated for cytokines (TNF-α, IL-6, IFN-γ, and IL-1β, respectively) determination by ELISA. Data were expressed as the mean ± SD (n = 4). *P < 0.05; NS, no significance.

High level of pro-inflammatory cytokines and uncontrolled inflammatory responses are the typical characteristics in lethal endotoxemia. As is shown in Fig. 2A–D, PLTP could suppress LPS induced TNF-α and IL-6 expression, suggesting that PLTP exerts anti-inflammatory effects in lethal endotoxemia. However, the levels of IFN-γ and IL-1β were not changed, indicating that the protective effects of PLTP did not involve in these pathways.

### Messenger RNA expression of pro-inflammatory cytokines induced by LPS is dramatically repressed by PLTP in macrophage

**Figure 3:**
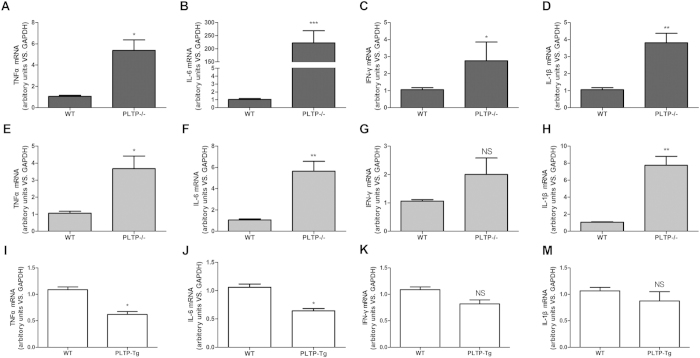
The expression of inflammatory cytokines in PLTP deficient macrophage induced by LPS. (**A–D)** Peritoneal derived macrophage (PDM) was stimulated with LPS (200 ng/mL) for 12 h. mRNA of TNF-α, IL-6, IFN-γ, and IL-1β was evaluated with real-time PCR (showed as Dark column). Expression levels of mRNA were indicated as fold difference compared with wild type group (WT). (**E–H)** Bone marrow derived macrophages (BMDM) from PLTP knockout mice (PLTP−/−) or WT were stimulated with LPS (200 ng/mL) for 12 h. mRNA of TNF-α, IL-6, IFN-γ, and IL-1β was evaluated with real-time PCR(showed as Gray column). Expression levels of mRNA were indicated as fold difference compared with WT. (**I–M)** BMDM from PLTP overexpressed mice or WT was stimulated with LPS (200 ng/mL) for 12 h. mRNA of TNF-α, IL-6, IFN-γ, and IL-1β was evaluated with real-time PCR(showed as Gray column). Expression levels of mRNA were indicated as fold difference compared with WT. Data were expressed as the mean ± SD (n = 4). *P < 0.05 VS. WT.; **P < 0.01 VS. WT.; ***P < 0.001 VS. WT.; NS, no significance VS. WT.

Macrophage is one of the key innate immune cells, which initiates inflammatory response via releasing pro-inflammatory cytokines. Moreover, the major source of active PLTP is macrophage. Therefore, to understand the exact role of PLTP on LPS triggered inflammatory responses, PDM or BMDM from WT or PLTP−/− were treated with LPS (200 ng/ml) for 12 h. As is shown in Fig. 3A–D, PLTP deficiency dramatically enhanced mRNA expression of TNF-α, IL-6, IFN-γ, and IL-1β induced by LPS in PDM. It is noteworthy that macrophage activation and accumulation stimulated by thioglycollate broth were observed during PDM collection^17^. Therefore, to obtain unactivated “resident” macrophage, we harvested BMDM from WT or PLTP−/− and carried out the same experiments. As is shown in Fig. 3E–H, PLTP deficiency significantly enhanced mRNA expression of TNF-α, IL-6, and IL-1β. Considering the cytokine expression profiles, we chose BMDM as an inactive macrophage model for the study^18^. To confirm the effects of PLTP on LPS stimulation, we conducted the same assay in BMDM of PLTP-Tg. As is shown in Fig. 3I–M, the expression of IL-6 and TNFα were decreased in PLTP-Tg 8 hours after LPS stimulation, while the expression of IFN-γ and IL-1β were not changed. Above findings strongly suggested that PLTP is necessary to repress LPS triggered inflammation in macrophage.

### PLTP represses the expression of pro-inflammatory cytokines induced by LPS

**Figure 4:**
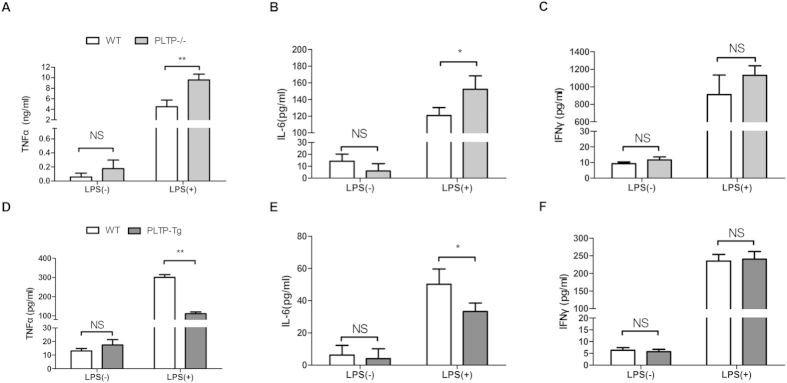
The effects of PLTP on pro-inflammatory cytokines level induced by LPS in macrophage. **(A–C)** After 200 ng/mL of LPS stimulation for 24 h, media from cultured BMDM of WT or PLTP−/− were harvested and conducted for TNF-α, IL-6, and IFN-γ quantization by ELISA. (**D–F)** After 200 ng/mL of LPS stimulation for 8 h, BMDM cultured media was harvested and conducted for TNF-α, IL-6, and IFN-γ quantization by ELISA. Data are expressed as the mean ± SD (n = 4). *P < 0.05; **P < 0.01; NS, no significance.

To confirm above findings, the levels of TNF-α, IL-6, and IFN-γ in cultured media were determined. Similar to mRNA results (Fig. 4A–C), PLTP deficiency significantly enhanced TNF-α and IL-6 in cultured media, while no change was found in IFN-γ. To exclude the compensation effects of PLTP deficiency, we also conducted the same assay in PLTP-Tg BMDM. As is shown in Fig. 4D–F, PLTP-Tg BMDM released less pro-inflammatory cytokines in 8 h after LPS stimulation. Our data proved that PLTP repressed the expression of pro-inflammatory cytokines induced by LPS. Considering that TNF-α and IL-6 are the target genes of activated NFκB in macrophage, we investigated whether PLTP attenuates LPS triggered NFκB activation^19^.

### PLTP deficiency or knockdown enhances LPS induced NFκB activation

**Figure 5:**
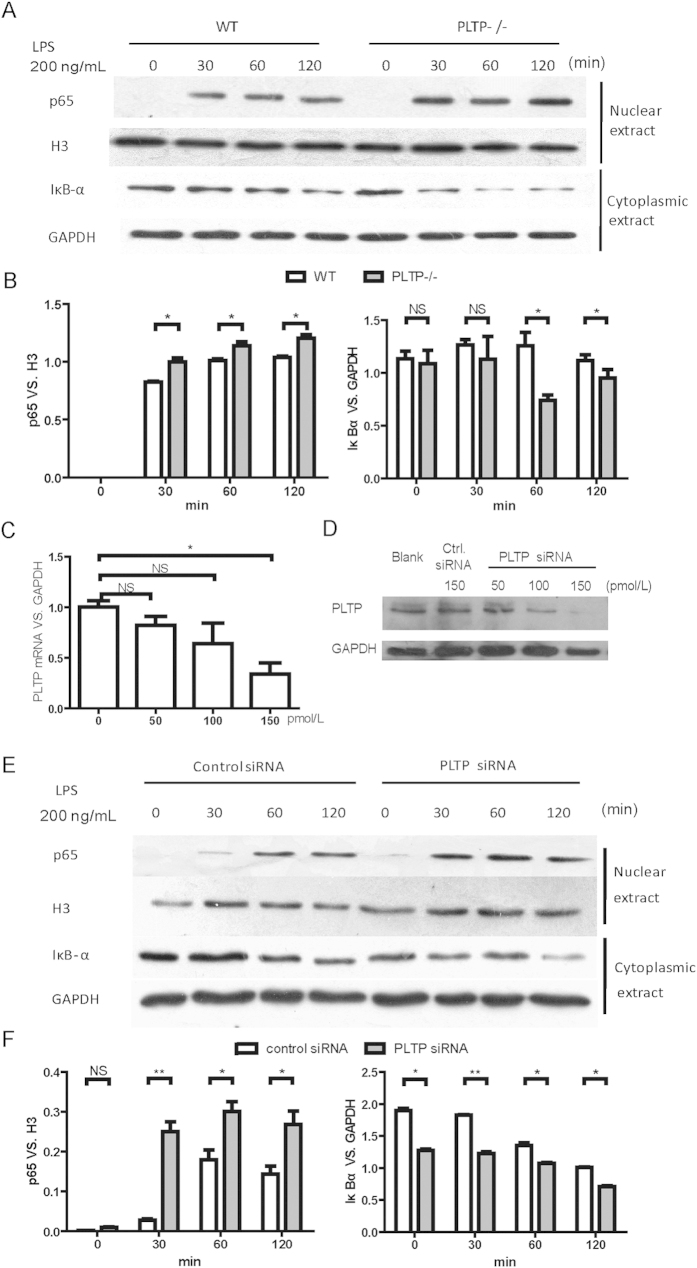
LPS induced NFκB activation in PLTP deficient macrophages and PLTP knockdown RAW264.7. Bone marrow derived macrophages (BMDM) or RAW264.7 were stimulated with LPS (200 ng/mL) for 0, 30, 60, 120 min. The nuclear and cytoplasmic extracts were determined with anti-p65 and anti-IκBα, respectively. Anti-histone 3 (H3) and anti-GAPDH antibodies were employed as nuclear and cytoplasmic protein loading controls, respectively. PLTP (**A)** Nuclear p65 and cytoplasmic IκBα in BMDM from wild type mice (WT) VS. PLTP knockout (PLTP−/−). (**B)** Densitometry analysis of nuclear p65 and cytoplasmic IκBα in BMDM. (**C**,**D)** the mRNA level and protein level of PLTP knockdown via siRNA (150 pmol/L for PLTP siRNA and control siRNA, respectively) transfection. (**E)** Nuclear p65 and cytoplasmic IκBα from control siRNA or PLTP siRNA treated RAW264.7. (**F)** Densitometry analysis of nuclear p65 and cytoplasmic IκBα in RAW264.7. These results are a representative of 3 independent experiments. *P < 0.05; **P < 0.01; NS, no significance.

We evaluated the role of PLTP in LPS induced NFκB activation in PLTP−/− BMDM and PLTP knockdown RAW264.7 by western blot (WB). As is shown in Fig. 5A,B, nuclear p65 level was significantly enhanced in PLTP−/− BMDM after LPS stimulation. Consistently, cytoplasmic IκBα degradation in PLTP−/− BMDM was enhanced in a time-dependent manner after LPS treatment. To confirm the above findings and exclude the compensation effects of PLTP deficiency, we also evaluated the NFκB activation in PLTP knockdown RAW264.7 induced by LPS. As is shown in Fig. 5C–F, PLTP knockdown enhanced the nuclear content of p65 stimulated by LPS dramatically. Furthermore, PLTP knockdown had significantly increased LPS induced cytoplasmic IκBα degradation. Consisted with the data in PLTP−/− BMDM, these results suggested that the macrophage derived PLTP was the main cause to repress NFκB activation induced by LPS.

### Macrophage derived PLTP does not activate STAT3-SOCS3 pathway

Recombinant PLTP showed an anti-inflammatory effect by activating ABCA1-JAK2-STAT3 pathway^14^. To clarify whether macrophage derived PLTP repressed NFκB activation via ABCA1-STAT3 pathway, we checked the level of pSTAT3 in nuclear and SOCS3 in cytoplasm, a downstream anti-inflammatory effecter of pSTAT3, in LPS treated macrophage with or without the presence of Glyburide, a chemical ABCA1 inhibitor. In this study, the level of pSTAT3 and SOCS3 were not activated either in PLTP knockout BMDM (supplementary -Figure-1A and B) or in PLTP knockdown RAW264.7 (supplementary-Figure-1C and D). Our findings proved that macrophage derived PLTP from WT BMDM did not activate STAT3-SOCS3 pathway.

### Macrophage derived PLTP attenuates LPS induced TNFα expression without the alteration of TLR4 abundance

**Figure 6:**
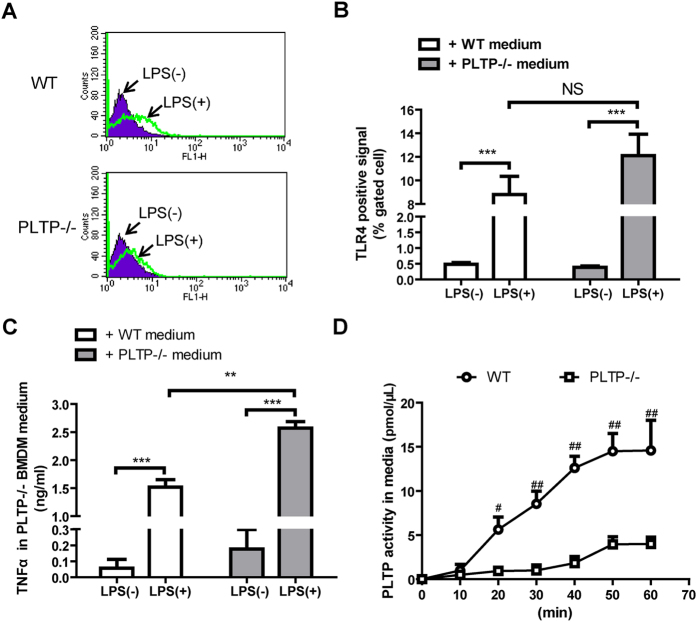
The role of macrophage derived PLTP on LPS induced TNFα expression and TLR4 activation. **(A**) BMDM with or without LPS stimulation were stained with 1 μg/mL TLR4-MD-2 complex antibody for 1 hour on ice, then washed with ice cold PBS for 3 times and then analyzed on Flow Cytometer. (**B**) Gated cell counting and analysis of positive signal. (**C**) 200 ng/ml LPS was incubated for 60 min with concentrated cultured media (CCM) from WT or PLTP−/− BMDM, and then the mixture were added into PLTP−/− BMDM for another 24 h culture. The medium was harvested for TNFα level determination by ELISA. (**D**) the CCM from WT or PLTP−/− BMDM was harvested for PLTP activity determination. Data are expressed as the mean ± SD (n = 4). *P < 0.05; NS, no significance.

As is shown in Fig. 5A,B, no difference was observed between WT and PLTP−/−, which indicated that PLTP expression could not affect the distribution and function of TLR4. Previous data strongly suggested that macrophage derived PLTP exerts its protective effects from LPS stimulation via a potential extracellular mechanism. Therefore, to clarify the role of macrophage secreted PLTP on LPS, the concentrated cultured medium (CCM) of macrophage were harvested for co-incubation with LPS before the treatment of BMDM. As is shown in Fig. 5C, PLTP−/− (CCM) treated LPS could induce higher level of TNFα compared with WT CCM treated LPS. The results indicated that PLTP−/− CCM treated LPS showed stronger pro-inflammatory effects compared with WT CCM treated LPS. To confirm the presence of PLTP in harvested medium, we also determined the phospholipid transfer activity of CCM. As is shown in Fig. 6D, PLTP activity was higher in CCM from WT BMDM compared with PLTP−/− BMDM. Our data elucidated that extracellular LPS neutralization might be the main cause of repression of NFκB activation by macrophage derived PLTP.

### PLTP binds to LPS and forms low cell toxic complex

**Figure 7:**
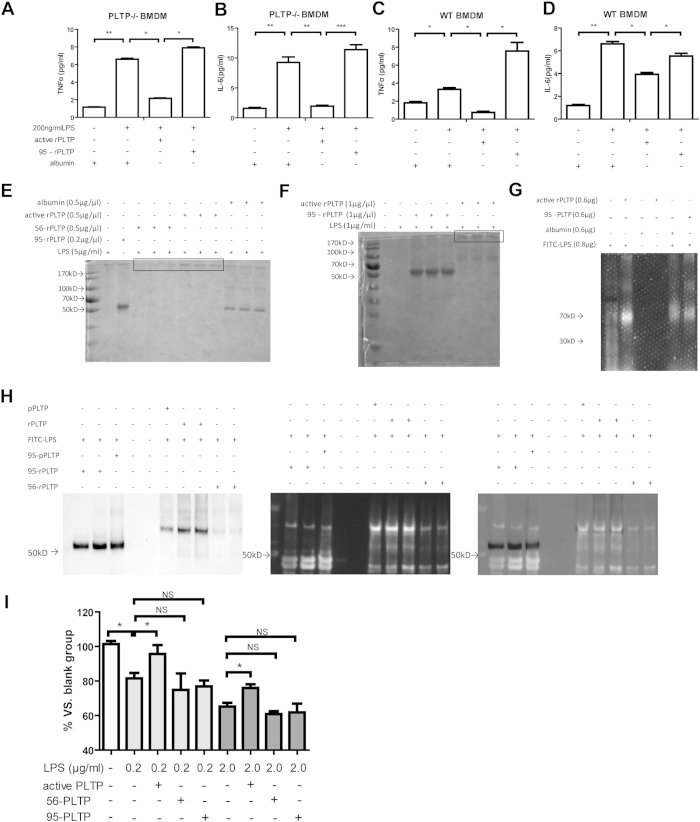
The binding capability of PLTP to LPS and the macrophage toxicity determination of PLTP-LPS complex. For co-incubation assay, active recombinant PLTP (rPLTP), rPLTP treated in 56 °C for 30 min (56-rPLTP), and rPLTP treated in 95 °C for 5 min (95-rPLTP) were incubated with LPS in a final volume of 50 μL for 60 min, respectively. The mixture was loaded in 10% native gel for electrophoresis, staining, immunoblot, and photographing. (See Materials and Methods for detail) (**A,B)** the levels of TNFα and IL-6 in media of PLTP−/− BMDM after the stimulation of rPLTP-LPS mixture or albumin-LPS mixture for 24 h, respectively. (**C,D)** the levels of TNFα and IL-6 in media in medium of WT BMDM after the stimulation of rPLTP-LPS mixture or albumin-LPS mixture for 24 h, respectively. (**E)** The rPLTP, 95-rPLTP, albumin, and 56-rPLTP were incubated with LPS, respectively. The mixtures were run in native gel and photographed after Coomassie blue staining. (**F)** The rPLTP, albumin, and 95-rPLTP were incubated with LPS, respectively. The mixture was run in native gel and photographed after Coomassie blue staining. (**G**) FITC conjugated LPS (FITC-LPS) was incubated with rPLTP and albumin, respectively. The mixtures were run in native gel and the fluorescence was photographed (Excitation: 488 nm, Emission: 525 nm). The full length gel was in supplementary-figure-2; (**H**) FITC-LPS was incubated with rPLTP, 56-rPLTP, 95-rPLTP, respectively. Purchased recombinant PLTP (pPLTP) and pPLTP treated in 95 °C for 5 min (95-pPLTP) were loaded as positive control and negative control for LPS incubation, fluorescence photographing, and WB analysis, respectively. Left panel, the WB results of co-incubated mixture, detected by PLTP antibody; intermediate panel, FITC signals detected by fluorescence photographing; right panel, the merge picture. The assays were conducted for 3 times and the whole length gels or blots were showed. (**I**) rPLTP/LPS mixture was co-incubated with RAW264.7 cells for 24 h and the cell viability was assayed by MTT. Data are expressed as the mean ± SD (n = 4 for experiments in (**A–D**) n = 3 for experiments in (**E–H**) n = 6 for experiments in (**I**)). *P < 0.05; **P < 0.05; ***P < 0.05.

To understand how PLTP neutralizes the macrophage toxicity of LPS *in vitro*, we determined pro-inflammatory effects of LPS pre-incubated with active or inactive recombinant PLTP (rPLTP). Compared with heat-inactived rPLTP treated LPS or albumin treated LPS, active rPLTP treated LPS could induce lower TNF-α and IL-6 expression in PLTP−/− BMDM, which strongly suggested that PLTP repressed LPS induced inflammation more effectively than albumin. (Fig. 7A,B) Considering that PLTP deficiency may cause certain genes expression compensatively, we also conducted the similar experiment in WT BMDM. As is shown in Fig. 7C,D, rPLTP could repress LPS induced TNF-α and IL-6 expression, while the levels of cytokines were much lower compared with the assay in PLTP−/− BMDM.

Above results strongly suggested that PLTP could bind to LPS and form low cell toxic complexes. To prove this, we separated the co-incubated mixture of PLTP and LPS with native gel. As is shown in Fig. 7E,F, most of active PLTP and 56 °C treated PLTP were consumed and large size complex were stacked in the borderline of running gel (indicated in the thin line box), while no obvious consumption of the albumin or the 95 °C denatured PLTP were observed. The results proved that the active PLTP and LPS may form the large size complex during co-incubation. To locate the LPS in the co-incubated mixture and to characterize the LPS binding capability to PLTP or albumin, the FITC labelled LPS (FITC-LPS) was employed in co-incubation system. As is shown in Fig. 7G, active PLTP could concentrate more FITC-LPS than albumin or 95 °C treated PLTP, suggesting that PLTP show a higher affinity to LPS than albumin. Furthermore, to identify the large size complex formed by LPS and PLTP, the co-incubated mixture of PLTP and FITC-LPS was determined by WB. A purchased PLTP (pPLTP) was loaded as positive control. As is shown in Fig. 7H, active PLTP collocated with LPS and formed large size complex, while 95 °C treated PLTP could not bind to LPS in the co-incubation system. In addition, consisted with the results in Fig. 7E, 56 °C treated PLTP could bind to LPS, whereas the complex bands were weaker compared with active PLTP, suggesting that a lower binding capability was displayed in the 56 °C treated PLTP. To clarify the toxicity of rPLTP/LPS, RAW264.7 cells were treated with pre-incubated complex of rPLTP/LPS for cell viability determination. As is shown in Fig. 7I, rPLTP (0.5 μg/ml) neutralized LPS and increased the cell viability challenged by different concentrations of LPS (0.2 μg/ml and 2.0 μg/ml, respectively), while no cell protection was observed in heat-treated PLTP groups. The results indicated that PLTP could bind to LPS and form low cell toxic complex in the co-incubation system.

## Discussion

Accumulated circulatory LPS and uncontrolled inflammatory responses are the main causes of individual death in lethal endotoxemia. Herein, we reported for the first time that: 1) plasma PLTP activity, but not HDL pool size, was critical for improving the individual survival rate in endotoxemia; 2) the expression of pro-inflammatory cytokines induced by LPS was suppressed by macrophage derived PLTP via the attenuation of NFκB activation; 3) STAT3-SOCS3 pathway was not activated by macrophage derived PLTP; 4) PLTP could bind to LPS and form low cell toxic complex *in vitro*. Our data highlighted the finding that PLTP is the primary extracellular protective protein to survival in lethal endotoxemia and to repress LPS induced inflammatory responses.

Previous observations supported that enlarging the plasma HDL pool size might characterize a therapeutic approach to the treatment of endotoxemia and sepsis^4^,^5^. Regarding their quality, HDL particles are highly heterogeneous and contain varying levels of anti-inflammatory agents and pro-inflammatory agents, which result in variation of the HDL function. Furthermore, HDL may lose its anti-inflammatory activities and transform into acute-phase HDL in endotoxemia or sepsis^20^. For this purpose, we focused on the proteins involving HDL metabolism. PLTP mediates lipid transferring among lipoproteins and promotes HDL remodeling. Moreover, the LPS neutralization capability indicated that PLTP exerts its potential protective roles in endotoxemia^6^. However, PLTP was still considered as a transfer protein mediating LPS to HDL particles other than a directive inflammatory suppressor *in vivo*.

Severe inflammatory responses are the main cause of organ failure and the individual death in endotoxemia. During acute or chronic inflammation, various acute-phase proteins, including PLTP, secreted by activated macrophage to participate in the regulation of inflammation, play an important role in innate immune response^8^,^9^,^11^,^20^. A previous study showed that extracellular PLTP (5 μg/ml) suppresses NFκB activation via ABCA1-JAK2-STAT3 pathway, whereas the source and concentration of PLTP are limited in local tissue where macrophage is found^14^. Furthermore, the mass of plasma PLTP was decreased, while the activity was increased in acute-phase patients, which suggested that the active PLTP, but not total PLTP plays the major protective role in local inflammation^9^. Therefore, to clarify the role of active PLTP in endotoxemia, we conducted the present study and found that PLTP displayed a strong protective effect on lethal endotoxemia in mice survival study.

Pro-inflammatory cytokines from macrophage initiates organ damage and aggravates the existing inflammatory responses in endotoxemia. Activated macrophages are polarized into two major activated subtypes termed M1 (classic activated by LPS or IFN) and M2 (alternative activated by IL-4 *et al*.)^21^. The cytokine expression profile induced by LPS indicated that the main subtype of BMDM is pro-inflammatory macrophage in this study. Activated macrophage is one of the major sources of pro-inflammatory cytokines, including TNF-α, IL-6, and IL-1β. Therefore, to understand the effect of PLTP on LPS activated macrophage, the LPS induced pro-inflammatory cytokine expression was also investigated. Consistent with the *in vivo* study, PLTP would repress the expression of pro-inflammatory cytokines induced by LPS in macrophage. Considering that the cytokines are the target genes of NFκB in macrophage, we determined the effects of PLTP expression on LPS induced by NFκB activation.

NFκB activation is the primary transcription pathway to mediate acute and chronic inflammatory responses^21^. The subsequent pro-inflammatory cytokines expression aggravates local tissue or cell damage in macrophage mediated inflammation. Inhibition of NFκB activation and suppression of pro-inflammatory cytokines expression are the vital targets of anti-inflammatory treatment^14^. Our findings indicated that PLTP was the key protein to repress NFκB activation in LPS induced inflammation. In addition, Vuletic S found that the active PLTP may reenter nucleus, suggesting that intracellular PLTP is a nucleocytoplasmic shuttling protein which is like a transcription factor^22^. Moreover, various acute-phase proteins or innate immunity proteins may interact with PLTP and form PLTP-protein complex in plasma, indicating that PLTP is a potential multifunctional protein which may interact with other cytoplasmic proteins^11^. Our results showed that PLTP repressed NFκB activation in BMDM and RAW264.7. According to previous studies and our findings, PLTP definitely showed the anti-inflammatory effects via the attenuation of NFκB activation. We also tried IFN-γ stimulated macrophage and found that PLTP do not affect IFN-γ induced NFκB activation. (Supplementary figure-3) Thus, the directed effects of PLTP on LPS were investigated. Furthermore, no STAT3-SOCS3 activation was observed in this study, suggesting that PLTP repressed LPS induced NFκB activation in an extracellular way.

Reported anti-inflammatory effects of recombinant PLTP consist of plasma LPS elimination and ABCA1-JAK2-STAT3 activation^6^,^14^. In this study, no ABCA1 mediated STAT3 activation by macrophage derived PLTP was observed, which is probably due to the concentration of PLTP in cultured medium which was not high enough to reach the threshold of ABCA1 activation. In addition, to further clarify the role of PLTP on LPS in medium, we studied the “LPS activity” treated with PLTP in PLTP−/− BMDM. The data suggested that the macrophage derived PLTP showed a uneglectable LPS neutralization effect in cultured medium.

TLR4 is the well-recognized LPS receptor which initiates NFκB activation. Certain genes involving lipid metabolism could attenuate NFκB activation via impairing TLR4 recruitment and reducing TLR4 abundance^23^. Considering that PLTP might be a potential phospholipid transporter of plasma membrane, we examined the TLR4 activation in membrane. No TLR4 change was observed either in baseline BMDM or LPS challenged BMDM, indicating that the alteration of TLR4 activation was not the main cause of NFκB activation. Recognizing that the membrane total TLR4 level alteration is not necessary for LPS induced activation, the accumulation of unneutralized LPS was considered to be the main cause of enhanced NFκB activation in low PLTP expression status. Therefore, we conducted the PLTP-LPS co-incubation assays. In this study, no bivalent cation was added in co-incubation system which meant that the major size of LPS polymer was approximately 20 kD^24^. The results indicated that PLTP could bind to free LPS molecules and form less toxicity complex. Consequently, our findings clarified that the LPS neutralization role of PLTP was the main cause of the protective effects of PLTP in endotoxemia. Previous reports characterized that PLTP could extract LPS from Gram-negative bacterial membranes and neutralize LPS^6^,^25^. Plasma PLTP might be one critical plasma protein to increase survival rate in lethal endotoxemia^12^. Enlightened by these findings, we conducted this study. Consistently, our data supported the statement that PLTP was an essential acute phage protein to suppress LPS induced inflammation *in vivo* and *in vitro* via the LPS neutralization capability.

To summarize, PLTP protected mice from the attack of accumulated circulatory LPS, and subsequently increased the survival rate in lethal endotoxemia via its LPS neutralization capability. The protection was not affected by HDL pool size. The findings also indicated that higher level of PLTP in acute infection or other inflammatory disease might be one of the key steps of host defensive responses.

## Materials and Methods

### Animals

PLTP−/− and PLTP-Tg were kindly obtained from Dr. Jiang^7^. Animals were on a homogenous C57BL/6 background (9 generation backcrosses). Experimental animals were housed in a temperature and humidity controlled room with a 12/12 h light–dark cycle. 8–10 week-age of male mice (N = 10) were used in this study. All experiments were approved by the laboratory animals’ ethical committee of Taishan Medical University and followed national guidelines for the care and use of animals.

### Antibodies and reagents

Antibodies against nuclear factor kappa-B (NFκB) p65 phosphorylated at serine 276 (Cat. No. ab31481), H3 (Cat. No. ab9485), and GAPDH (Cat. No. ab9485) were purchased from Abcam (Cambridge, MA, USA). Antibodies against apolipoprotein AI (apoAI, Cat. No.sc-80551), IκBα (Cat. No. sc-1643) purchased from Santa Cruz (Dallas, Texas, USA). ELISA kits of mouse IL-6 (Cat. No. m6000b), interferon γ (IFN-γ, Cat. No. mif00), and tumor necrosis factor α (TNF-α, Cat. No. mta00) were purchased from R&D (Minneapolis, MN, USA). Antibodies against STAT3 (Cat. No. 4904), STAT3 phosphorylated at tyrosine 705 (Cat. No. 9131), and suppressor of cytokine signaling 3 (SOCS3) (Cat. No. 5206S) were purchased from Cell signaling technology (Danvers, MA, USA). LPS (Cat. No. L2880), thioglycollate broth (Cat. No. 70157), recombinant tumor necrosis factor α (Cat. No. T0157), Glyburide (Cat. No. G2539), LPS from *E. Coli* O55:B5 (Cat. No. L2880), LPS-FITC from *E. Coli* O55:B5 (Cat. No. F8666), PLTP siRNA, and control siRNA were purchased from Sigma-Aldrich (St. Louis, MO, USA). TLR4-MD-2 complex Antibody (Cat. No. 53–9041) was purchased from eBioscience. Fetal bovine serum (FBS), 3-(4,5-Dimethylthiazol-2-yl)-2,5-Diphenyltetrazolium Bromide (MTT), and lipofectamine 2000 transfection kit were purchased from Life technology (Grand Island, NY, USA). Complete protease inhibitor cocktail tablets (Roche 05892970001) and PhoStop cocktail (Roche 04906845001) were purchased from Roche (Schweiz, Germany). NBD-labelled phosphoethanolamine (NBD-PE, N-360) was purchased from Molecular Probe (Life technologies, USA). Recombinant PLTP was kindly presented by Dr. Jiang. Control recombinant PLTP (Cat. No. 4918-PL/CF) was purchased from R&D (R&D system, USA).

### LPS injection

LPS was suspended in endotoxin-free, 0.15 M sodium chloride and handled following reported method^12^. The LPS was injected intraperitoneally into mice (5 mg/kg body weight for PLTP−/−, and 6 mg/kg body weight for WT or PLTP-Tg; single doses).

### Plasma lipid analysis

Lipoprotein profiles were obtained by fast protein liquid chromatography (FPLC). Briefly, 100 μL pooled plasma was loaded onto a SuperoseTM 6 column (10/300) connected with the ÄKTA FPLC on purfier-900 system to separate the lipoprotein fractions, eluting with mobile phase (0.15 M NaCl, 0.01 M Na2HPO4 and 0.1 M EDTA, pH 7.5) at a flow rate of 0.3 ml/min. 50 fractions (0.5 ml each) of eluate were collected^26^. Cholesterol content from each fraction was determined.

### Cell culture, transfection, and PLTP-LPS co-incubation

Peritoneal derived macrophage (PDM) was collected following the previous methods^17^. To obtain bone marrow derived macrophage (BMDM), the marrow from femur and tibia was cultured for 7–10 days in complete culture medium (20% FBS in DMEM) supplemented with 20% L929 cell medium to provide macrophage colony-stimulating factor (M-CSF) and induced the differentiation of harvested cells into macrophages^27^. RAW264.7 was cultured following the methods reported by us^28^,^29^. All the serum employed in this study was LPS free fetal bovine serum (Gibco, 16000-044, Beijing, China). The cell transfection was performed following the manufacturer’s instructions of lipofectamine 2000. For LPS stimulation or intercellular PLTP assay, all cultured medium was replaced by serum-free DMEM prior to the coming experiments. Cultured medium for PLTP activity assay was harvested and concentrated with ultra 0.5 centrifugal filter (3 kD, Amicon). The co-incubation of LPS and CCM were carried out following the method reported previously with small modification^6^. Briefly, LPS was incubated alone or with 10–20 μl CCM in a final volume of 50 μl D-Hanks buffer solution (Hyclone, H1040, Beijing, China) at 37 °C for 60 min. The LPS-CCM mixtures were added in PLTP−/− BMDM as stimulation. Recombinant PLTP (0.6 μg) and LPS (0.8 μg) were diluted with D-Hanks buffer solution before co-incubation, respectively. The final concentration of rPLTP was 0.6 μg/ml after the addition of cultured media of PLTP−/− BMDM or WT BMDM. For FITC-LPS and PLTP co-incubation and WB detection, the loading amounts of PLTP (including rPLTP, 56 °C treated rPLTP, 95 °C treated rPLTP, and purchased PLTP) and FITC-LPS were 20 μg and 60 μg, respectively.

### RNA interference

PLTP knockdown in RAW264.7 was carried out following the instruction of transfection kit. Following the pretest of RNA interference, the 150 pmol/L of PLTP siRNA and control siRNA were used.

### Toxicity of rPLTP/LPS determination

The toxicity of rPLTP/LPS complex was determined by MTT assay following the instruction of MTT kit.

### RNA extraction and real-time PCR

Total RNA extraction, reverse transcription, and real-time PCR analysis were described previously^30^,^31^. Primer sequences for real-time PCR were listed in Table 1^32^.

### Protein isolation, Electrophoresis, and WB

Proteins were isolated using cell lysis buffer with complete protease inhibitor cocktail tablets and PhoStop cocktail, which preserves phosphorylated sites in the presence of protease inhibitors. Total protein extraction from cultured cells or total plasma (for apoAI content assay) and WB analysis were described previously^28^. Densitometry analysis was conducted using Image-Pro Plus software version 6.0 (Media Cybernetics Corp, Bethesda, MD, USA). For mobility comparison of protein-LPS complex, the mixtures were loaded in 10% native gel for separation^33^. The Gel was stained with Coomassie brilliant blue and photographed.

### ELISA

Content of TNF-α, IL-6, and IFN-γ in cultured media were assessed by ELISA kits following the manufacturer’s instructions, respectively. The absorbance value was obtained from multifunctional multiple microplate reader (Infinity F200, TECAN, Austria).

### Cell surface receptor analysis

BMDM was stained with 1 μg/ml TLR4-MD-2 complex antibody for 60 min on ice, washed with ice cold PBS for 3 times and then analyzed on Flow Cytometer (BD FACS Calibur).

### PLTP activity assay

PLTP activity was determined following the method reported previously with brief modification^34^. Before the assay, the donor and acceptor were prepared respectively. The donor liposome labeled with NBD-PE was in a stable and self-quenched status before use. The donor (3 μl) and acceptor (3 μl) with plasma (3–5 μl) or concentrated cultured media (3–10 μl) were combined in a final volume of 100 μl TNE (10 mM Tris, 0.15 M NaCl, 2 mM EDTA, pH = 7.4) at 37 °Cin a 96-well black microplate to allow the transfer of NBD-PE mediated by PLTP, and the fluorescence was detected every 10 minutes using a multifunctional microplate reader (Infinity F200, TECAN, Austria, EX465nm/EM535nm). The transfer rate was expressed as pmol/μl/min.

### Statistical analysis

All the experiments were repeated three or four times and GraphPad Prism software was used for statistical analysis. Data were typically expressed as mean ± S.D. Data between two groups were analyzed by Student’s t test. Survival rates were analyzed by the Kaplan-Meier method and compared using the X^2^ test. A statistically significant difference was assumed at P < 0.05^12^.

## Additional Information

**How to cite this article**: Yu, Y. *et al*. The binding capability of plasma phospholipid transfer protein, but not HDL pool size, is critical to repress LPS induced inflammation. *Sci. Rep.* **6**, 20845; doi: 10.1038/srep20845 (2016).

## Supplementary Material

Supplementary Information

## Figures and Tables

**Table 1:** Primers used for real-time PCR analysis.

Gene	Primer	Sequence (from 5′ to 3′)
IL-6	Sense	ACCACGGCCTTCCCTACTTC
	Anti-Sense	CTCATTTCCACGATTTCCCAG
TNF-α	Sense	CTGTAGCCCACGTCGTAGC
	Anti-Sense	GGTTGTCTTTGAGATCCATGC
IFN-γ	Sense	GCTCTGAGACAATGAACGCTAC
	Anti-Sense	TCTTCCACATCTATGCCACTTG
IL-1β	Sense	ACTGTTTCTAATGCCTTCCC
	Anti-Sense	ATGGTTTCTTGTGACCCTGA
GAPDH	Sense	TGACGTGCCGCCTGGAGAAA
	Anti-Sense	AGTGTAGCCCAAGATGCCCTTCAG
