# Supplementary material for: The binding capability of plasma phospholipid transfer protein, but not HDL pool size, is critical to repress LPS induced inflammation
Source: Sci Rep. 2016 Feb 9;6:20845. doi: 10.1038/srep20845 (PMC4746621; doi:10.1038/srep20845)

The binding capability of plasma phospholipid transfer protein, but not HDL pool size, is critical to repress LPS induced inflammation

Yang Yu[1,4#], Yingjie Cui[1#], Yanan Zhao[1#], Shuai Liu[1], Guohua Song[1,4], Peng Jiao[1], Bin Li[1], Tian Luo[1], Shoudong Guo[1], Xiangjian Zhang[3], Hao Wang[1], Xian-Cheng Jiang[2], Shucun Qin[1 *]

[1]Key Laboratory of Atherosclerosis in Universities of Shandong and Institute of Atherosclerosis, Taishan Medical University, Taian, China, 271000

[2]Department of Anatomy and Cell Biology, SUNY Downstate Medical Center, New York, USA, 11203

[3]Hebei Collaborative Innovation Center for Cardio-cerebrovascular Disease and Hebei Key L aboratory of Vascular Homeostasis, Shijiazhuang, China, 050000

[4]School of Basic Medicine, Taishan Medical University, Tai'an, China, 271000

Supplementary-FIGURE-1. STAT3-SOCS3 pathway was not activated in BMDM or RAW264.7 cells induced by LPS. WT or PLTP-/- BMDM was culture for 2 days without refreshing culture media for secreted PLTP accumulation. Glyburide was added to inactive ATP binding cassette A1 (ABCA1) 30min before LPS stimulation. The nuclear extract was probed with anti-phosphorylated signal transducer and activator of transcription 3 (STAT3). Histone 3 (H3) were employed as nuclear protein loading controls. **A,** upper panel, pSTAT3 contents in nucleus treated with Glyburide or/and LPS; lower panel, densitometry analysis of pSTAT3 contents. **B,** upper panel, SOCS3 contents in cytoplasm; lower panel, densitometry analysis of SOCS3 contents. PLTP siRNA or control siRNA transfected RAW264.7 cells were stimulated with LPS for 24h, the nuclear and cytoplasmic extracts were detected with anti-pSTAT3 and anti-SOCS3 respectively. **C,** Nuclear pSTAT3 content and densitometry analysis. **D,** Cytoplasmic SOCS3 content and densitometry analysis. These results are a representative of 3 independent experiments. *, $P < 0.05$, NS, no significance.

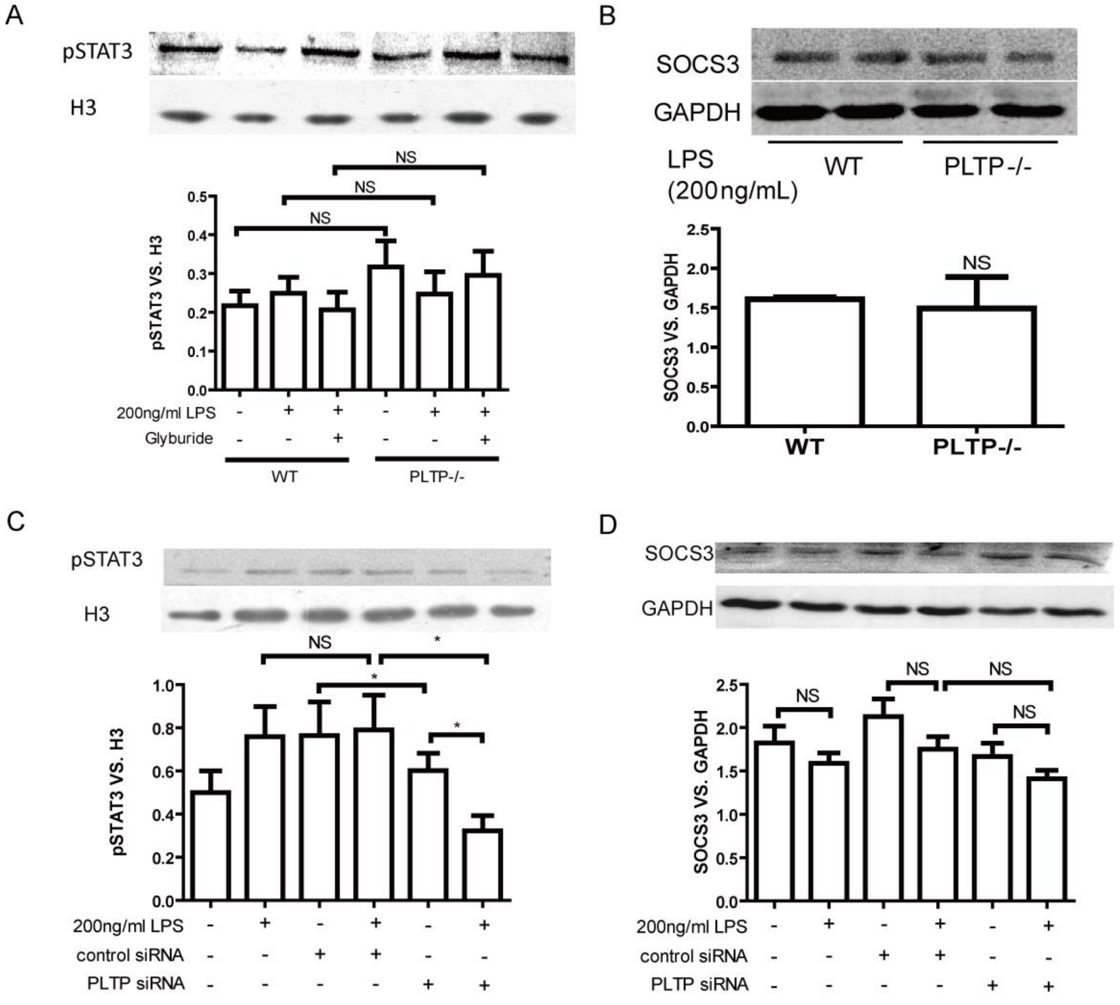

Supplementary-FIGURE-2. The full length gels of PLTP and FITC conjugated LPS were listed below. FITC conjugated LPS (FITC-LPS) was incubated with rPLTP and albumin, respectively. The mixtures were loaded in native gel. The fluorescence of FITC-LPS was photographing (Excitation: 488nm, Emission: 525nm). The assays were conducted for 3 times and the representative whole length gel was shown.

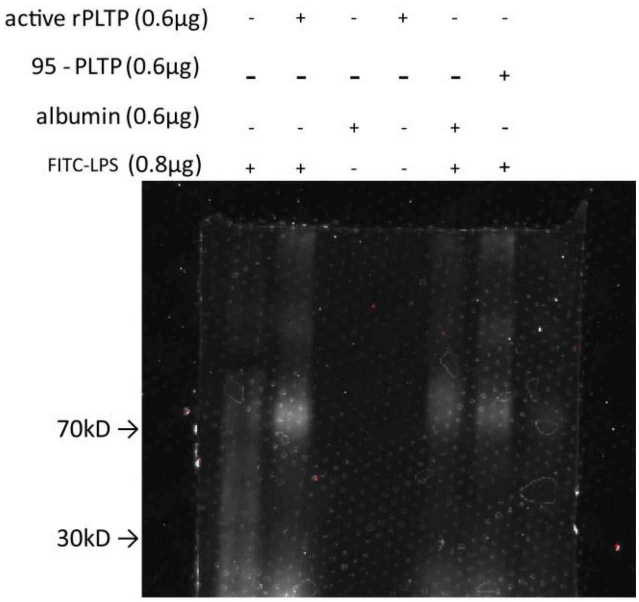

Supplementary-FIGURE-3. IFN-γ induced NFκB activation was not changed in PLTP deficient macrophages and PLTP knockdown RAW264.7. Bone marrow derived macrophages (BMDM) or RAW264.7 were stimulated with IFN-γ(100ng/mL) for 0, 30, 60, 120min, respectively. The nuclear and cytoplasmic extracts were determined with anti-p65 and anti-IκBα, respectively. Anti-histone 3 (H3) and anti-GAPDH antibodies were employed as nuclear and cytoplasmic protein loading controls, respectively. **A,** Nuclear p65 and cytoplasmic IκBα in BMDM from wild type mice (WT) VS. PLTP knockout (PLTP-/-). **B,** Densitometry analysis of nuclear p65 and cytoplasmic IκBα in BMDM. **C,** PLTP knockdown was conducted via siRNA (150pmol/L for PLTP siRNA and control siRNA, respectively) transfection. Nuclear p65 and cytoplasmic IκBα from control siRNA or PLTP siRNA treated RAW264.7. **D,** Densitometry analysis of nuclear p65 and cytoplasmic IκBα in RAW264.7. These results are a representative of 3 independent experiments. *, $P < 0.05$; NS, no significance.

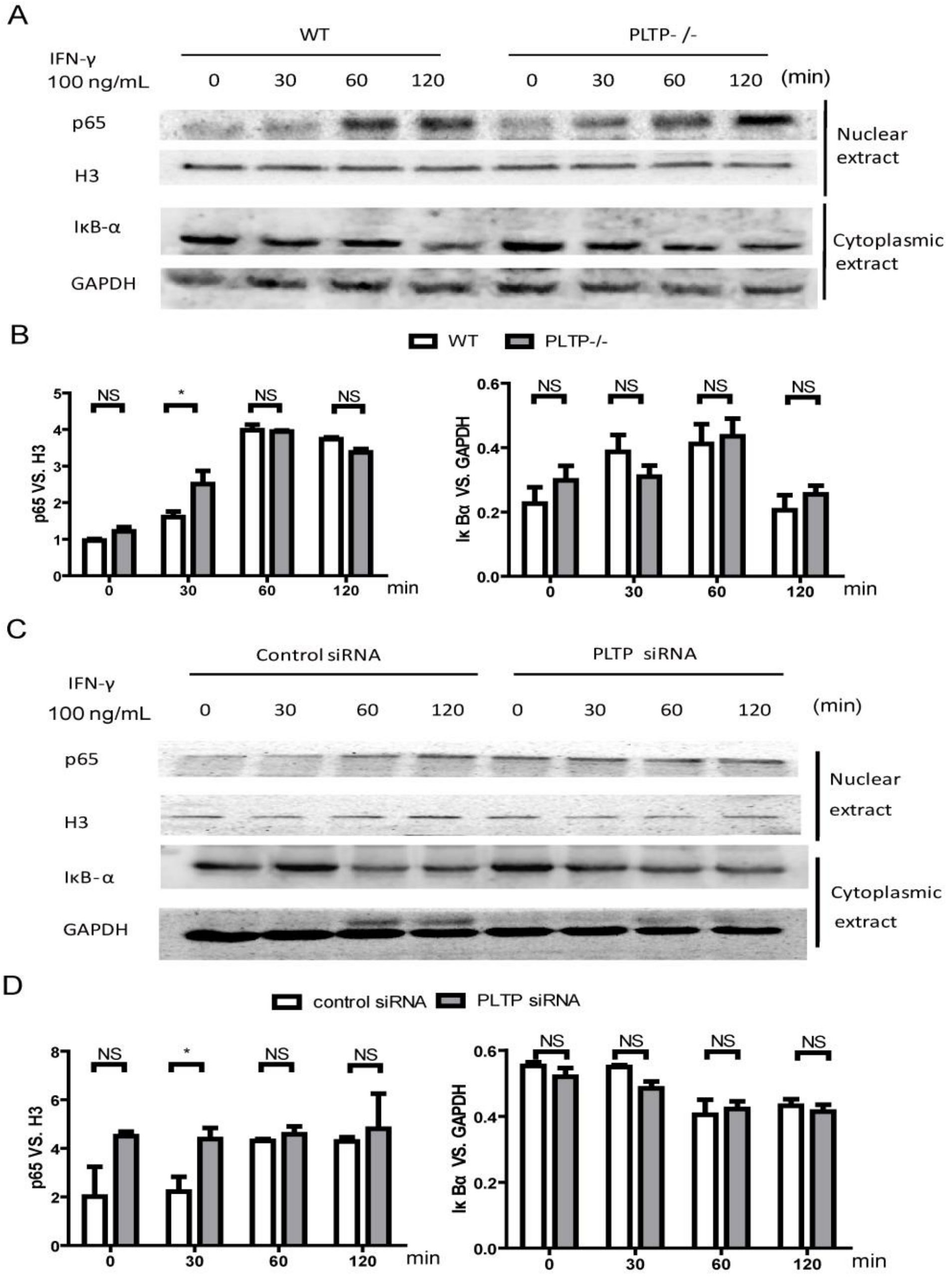

Supplement: Supplementary Information [file srep20845-s1.pdf]
